# Phylogeny based discovery of regulatory elements

**DOI:** 10.1186/1471-2105-7-266

**Published:** 2006-05-22

**Authors:** Jason Gertz, Justin C Fay, Barak A Cohen

**Affiliations:** 1 Department of Genetics, Washington University School of Medicine, 4444 Forest Park Parkway, St. Louis, MO 63108, USA

## Abstract

**Background:**

Algorithms that locate evolutionarily conserved sequences have become powerful tools for finding functional DNA elements, including transcription factor binding sites; however, most methods do not take advantage of an explicit model for the constrained evolution of functional DNA sequences.

**Results:**

We developed a probabilistic framework that combines an HKY85 model, which assigns probabilities to different base substitutions between species, and weight matrix models of transcription factor binding sites, which describe the probabilities of observing particular nucleotides at specific positions in the binding site. The method incorporates the phylogenies of the species under consideration and takes into account the position specific variation of transcription factor binding sites. Using our framework we assessed the suitability of alignments of genomic sequences from commonly used species as substrates for comparative genomic approaches to regulatory motif finding. We then applied this technique to *Saccharomyces cerevisiae *and related species by examining all possible six base pair DNA sequences (hexamers) and identifying sequences that are conserved in a significant number of promoters. By combining similar conserved hexamers we reconstructed known cis-regulatory motifs and made predictions of previously unidentified motifs. We tested one prediction experimentally, finding it to be a regulatory element involved in the transcriptional response to glucose.

**Conclusion:**

The experimental validation of a regulatory element prediction missed by other large-scale motif finding studies demonstrates that our approach is a useful addition to the current suite of tools for finding regulatory motifs.

## Background

The central assumption of comparative genomics is that functional sequences evolve under constraints while non-functional sequences evolve neutrally. This simple assumption underlies several useful algorithms that identify coding genes [1,2], non-coding RNAs [3-5], and cis-regulatory sites [6-11]. However, current methods for detecting cis-regulatory sites could be enhanced significantly by addressing two major issues. First, most transcription factor binding site (TFBS) analyses treat the species under consideration as independent, ignoring the underlying phylogeny that connects the species with each other. Second, comparative genomic analyses should incorporate known information about how functional sequences evolve. For example, gene finders have specific models for coding regions, splice donor and splice acceptor sites. Similar models of TFBS evolution should be incorporated into motif finders. We therefore developed a method to find TFBS in the genomes of related species that accounts both for the evolutionary relationships between the species under consideration and provides an explicit model for TFBS evolution based on weight matrix models [12] of known cis-regulatory motifs.

Three algorithms that incorporate phylogenetic information have recently been presented [13-15]. A major difference between the three methods is the underlying evolutionary model used to take into account the phylogenetic relationships of the species under consideration. EMnEM [14] uses a Jukes-Cantor model [16] in which the substitution rate inside the regulatory element is fixed ignoring the positional variation of the motif. PhyME [13] and PhyloGibbs [15] use an evolutionary binding site model proposed by Sinha et al. [17] which is similar to Felsenstein's molecular evolution model [18]. This model combines binding site specificity into the substitution rates. As a natural progression we use a more detailed HKY85 model [19], which allows us to incorporate binding site specificities as well as differences in transition and transversion rates.

Another property that separates motif finding algorithms is how they search the space of possible motifs. EMnEM and PhyME use Expectation-Maximization to find an optimal binding site, while PhyloGibbs uses a Gibbs sampling approach. Both motif search methods are designed and most commonly used to analyze sets of co-regulated genes. However not all TFBS can be found in this way because we lack sets of co-regulated genes for every transcription factor. Our method uses an exhaustive search technique that does not require an enriched set of genes and is therefore well suited to look for regulatory elements across all intergenic regions in the genome, without additional information on co-expression, functional annotation, or DNA:protein interactions.

Other comparative genomics methods, including those proposed by Cliften et al. [11] and Kellis et al. [10], have been implemented that search the genome exhaustively for regulatory elements. Our approach adds new information by considering the underlying phylogeny while exhaustively searching all hexamers for conserved sequences across all intergenic regions. By combining these hexamers we created putative regulatory motifs. We experimentally tested one motif prediction and found it to be a regulatory element that activates gene expression in response to glucose.

## Results

### Evaluating binding site conservation

Sequences that match TFBS are often preserved in the promoters of closely related species simply because there has not been enough time for the sites to decay, and not because they are functional. Therefore it may be beneficial to weight the significance attributed to conserved sequences in proportion to the phylogenetic distance between the species in which the sequences are found. To create a probability-based method that incorporates the evolutionary relationships among species, we applied molecular evolution models.

Molecular evolution models are used to analyze base substitutions between related species. For any given sequence in a multiple alignment taken from different species we determine whether the pattern of substitutions better fits a neutral model of evolution or a conserved model of TFBS evolution. An HKY85 [19] nucleotide substitution model underlies both our neutral and conserved models of TFBS evolution. The two models are identical except that in the neutral model genomic base frequencies are used as the equilibrium base frequencies whereas in the conserved TFBS model position specific base frequencies derived from weight matrix models of specific TFBS are used. In concept, substitutions in a conserved motif are allowed to occur at the neutral rate as long as they do not disrupt the TFBS. By comparing likelihoods between the two models over the entire binding site we determine whether the conserved model is a significantly better fit than the neutral model [20]. Likelihood ratio tests have been used effectively in a number of diverse situations and remain a powerful tool for testing simple hypotheses [21-24]. Our comparison of likelihoods, which is similar to a likelihood ratio test, enables us to incorporate phylogenetic distances and position specific motif variation into a probabilistic framework.

### Properties of the method

To determine the properties of this new approach we performed searches for conserved sequences in simulated intergenic regions. We simulated groups of orthologous promoters from different numbers of species at different evolutionary distances, which contained either conserved or neutral instances of a binding site (see Methods). We simulated over one million neutral binding sites in a total of over one billion base pairs. We used the Chi-Squared distribution as an approximation to our expected distribution of likelihood comparisons and set the significance cutoff at an FDR of 0.1% (see Methods). Using these criteria we did not observe a single false positive, an instance of a neutral binding site being called significantly conserved by the likelihood comparison. The results from these simulated data suggest that the method has high specificity with a false positive rate less than 1 in 10^6^.

**Figure 1:**
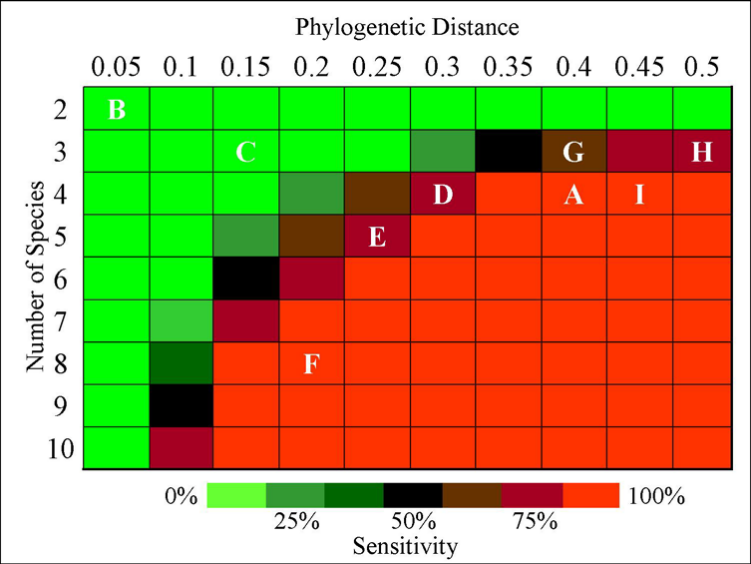
Sensitivity of the likelihood comparison on simulated data. Colors represent method's sensitivity with different numbers of species at different evolutionary distances (measured in substitutions per site). Letters represent approximate placement of commonly compared organisms. A: Yeast species used (96.964 % sensitivity) B : Human – Chimp (0 %) C : Human – Mouse – Rat (0 %) D : Human – Mouse – Rat – Chicken (83.489 %) E : Human – Mouse – Rat – Chicken – Dog (89.536 %) G : *T. nigroviridis – T. rubripes – D. Rerio *(68.372 %) H : *C. elegans *– *C. brigssae *– *C. remanei *(86.561 %) F : Human – Baboon – Cat – Dog – Cow – Pig – Mouse – Rat (98.761 %) I : *D Melanogaster – D. simulans – D. yakuba – D. psuedoobscura *(98.564 %).

The sensitivity, or ability to find conserved binding sites, of the method is affected by the number of species under consideration as well as the evolutionary distance between them, measured in substitutions per site. The addition of species and an increase in evolutionary distances both add to the sensitivity of the method. In Figure 1, some commonly compared organisms are plotted to demonstrate the theoretical sensitivity of these different data sets. For instance, with the *Saccharomyces *genomes under consideration in this study we achieve a sensitivity of 97%. Notably, alignments of human, mouse and rat do not contain enough statistical power to determine significantly conserved binding sites with high specificity. Most of the occurrences of TFBSs in these alignments are expected to be selectively neutral making the identification of those under purifying selection difficult. Addition of the chicken genome adds the resolution necessary to detect regulatory elements in human-mouse-rat alignments. These results are in good agreement with the theoretical analysis of Eddy [25]. One caveat of these simulations is that we assume perfect alignments at every evolutionary distance. In practice increasing the evolutionary distance between species increases the chances of generating faulty alignments that would decrease the sensitivity of the method. Since our results suggested that the method should work on the related yeast species, we decided to determine if our approach could be used to identify functional regulatory elements shared among these genomes.

**Figure 2:**
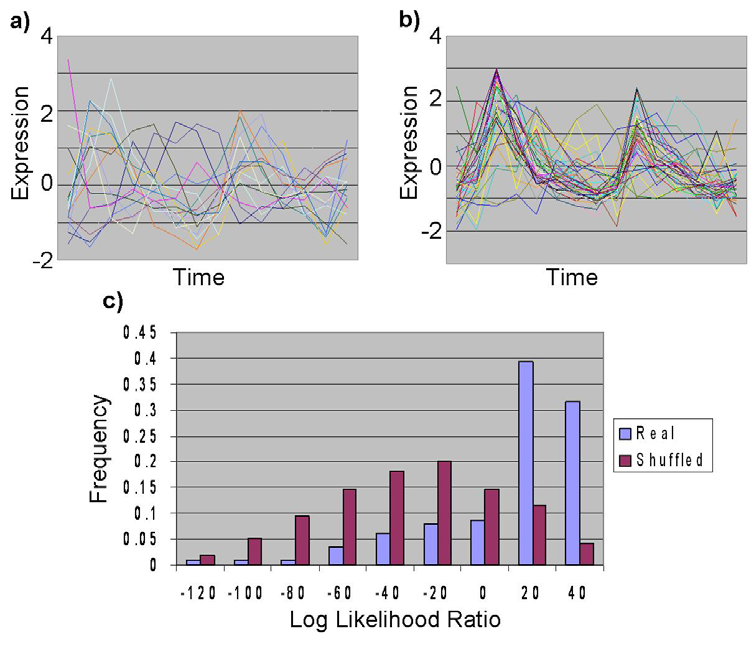
Cell cycle expression profiles for genes with **(a) **negative log likelihood ratio (better fit to the neutral model) MCB sites and **(b) **positive log likelihood ratio MCB sites in their promoter. The *x*-axis represents experimental timepoints through the cell cycle, and the *y*-axis represents relative gene expression levels (*Z*-scores). **(c) **Log likelihood ratios of the true MCB motif (blue) and shuffled MCB motifs (purple).

We first determined whether our method of scoring conserved sequences identifies functional instances of known motifs. Using the likelihood comparison on intergenic alignments [10] we classified all occurrences of MCB sites as either neutral or conserved. MCB sites are bound by the transcription factors Mbp1 and Swi6 which activate gene expression in the G1 phase of the cell cycle [26]. We plotted the expression levels, during a cell cycle time course [27], of genes whose promoters contained MCB sites that fit the neutral (Figure 2A) or the conserved (Figure 2B) models. Genes with neutral MCB sites showed random expression through the cell cycle while genes with conserved MCB sites displayed a strong expression pattern with two peaks corresponding to activation in the G1 phase of the cell cycle. These results suggest that our method may be able to distinguish functional from non-functional occurrences of TFBS.

We compared the output of our algorithm using either the true MCB weight matrix or a matrix in which the columns of the MCB model had been randomly shuffled. Although this shuffled model contains the same base frequencies and the same information content as the true MCB model, it does not represent any known TFBS. Using the true MCB site greatly enriched for positive log likelihood ratios (Figure 2C) (>70% of instances), indicating a better fit to the conserved model than the neutral model, while the random DNA motifs had more variation in log likelihood ratios with more negative log ratios. The observation that true regulatory elements have a very different distribution of log likelihood ratios than random DNA motifs led us to investigate whether the likelihood comparisons could identify novel transcription factor binding sites.

### Significantly conserved hexamers

**Figure 3:**
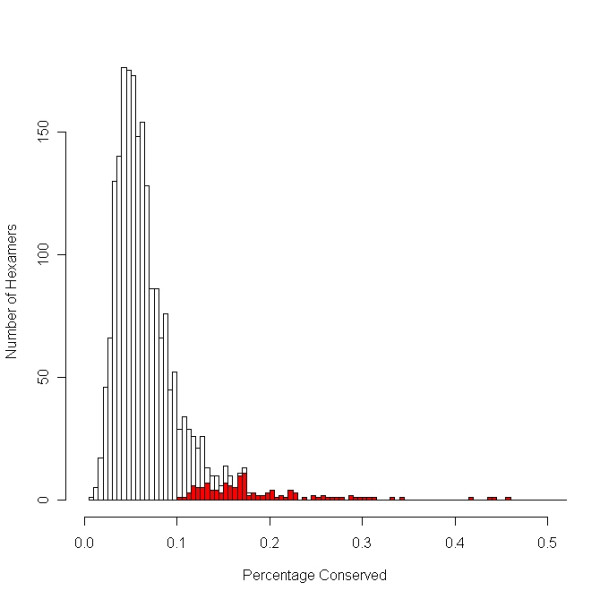
Histogram of percentage of significantly conserved occurrences for each hexamer. Hexamers with an unexpectedly high fraction (χ^2 ^≥ 10.828, *P *≤ 0.001) of conserved instances are shown in red.

To find new regulatory elements we decided to take an exhaustive approach in order to efficiently search the whole genome for regulatory elements. We therefore tested all six base pair DNA sequences (hexamers). For any particular hexamer, we identified all instances of that sequence in intergenic alignments and tested them using the likelihood comparison. We recorded the number of significantly conserved sites and the number of neutral sites for each hexamer to identify sequences in which a high fraction of the occurrences were scored as conserved. The assumption is that hexamers that are conserved often are likely to be part of a transcription factor binding site. The percentages of conserved sites for each hexamer are plotted in Figure 3. The mean fraction conserved was 0.0697 with a standard deviation of 0.0447. This suggests that most intergenic sequences are not significantly conserved; however, there is large variability in the amount of conservation between hexamers. The three most often conserved hexamers (CGGGTA, CCGGGT, GGGTAA) all match the Reb1 binding site showing that the most conserved intergenic sequences are indeed cis-regulatory sites.

We used a Chi-squared test (see Methods) to determine which hexamers were conserved in a higher fraction of intergenic regions than expected by chance. The resulting list consisted of 218 hexamers whose number of conserved instances differed from expected with *P *< 0.001 (shown in red in Figure 3) (see Additional file 1). There is no strict percentage cutoff corresponding to highly conserved hexamers. This is due to the variability in the number of intergenic occurrences for each sequence. Of these 218 conserved hexamers, 124 (57%) matched known binding sites, leaving 94 hexamers that were not correlated with known motifs.

We used a simple alignment approach (see Methods) to combine significant hexamers into motifs. From the starting 218 hexamers we derived 66 alignments corresponding to possible transcription factor binding site matrices. A total of 33 alignments matched 17 different known binding sites. This indicates that the false positive rate of our motif reconstruction method is at most 50%. However, this estimate assumes that all of the motif predictions that do not match known binding sites are incorrect, which is unlikely to be the case. None of the other 33 alignments (see Additional file 2), that did not match known motifs, matched previous predictions made by Kellis et al. [10], Cliften et al. [11] or Harbison et al. [28]; all recent high-throughput searches for cis-regulatory motifs based on comparative genome analyses and genome-wide chromatin immunoprecipitation (ChIP) data.

**Figure 4:**
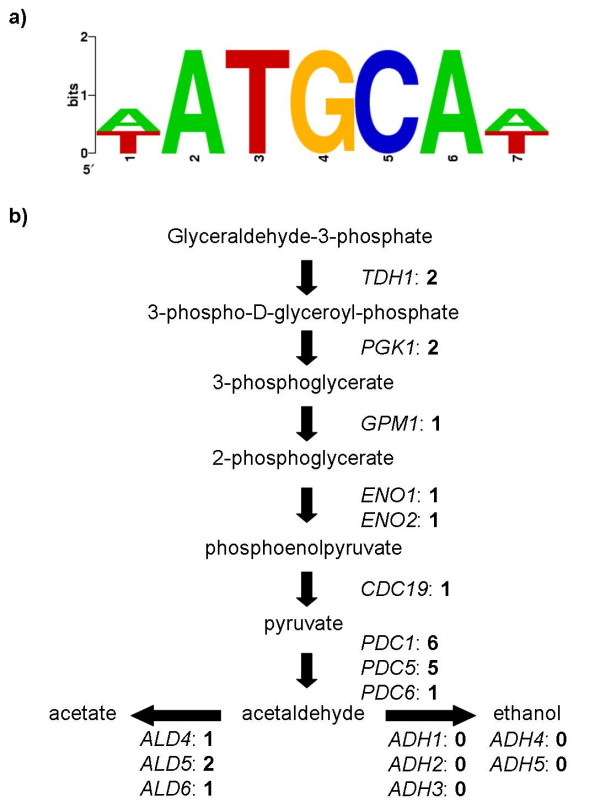
(a) Sequence logo [41] of putative regulatory motif. **(b) **Subsection of the glucose fermentation pathway. Numbers represent the occurrences of the putative motif in the gene's promoter.

To maximize the power of our approach we used each of the 66 new weight matrix models, we derived by combining conserved hexamers, in genome-wide searches for conserved sequences. For each of the 66 new weight matrices we constructed a corresponding HKY85 model and used it to search for sequences with positive log likelihood scores. To focus our attention on the most promising binding site predictions we looked for functional enrichment of genes with conserved motifs present in their promoters. The most promising motif (shown in figure 4A), based on being found upstream of genes with a statistical over representation in a functional class, was significantly over represented in the promoters of genes involved in carbohydrate utilization (*P *= 2.63 × 10^-5^). In figure 4B a subsection of the glucose utilization pathway in yeast is shown along with the number of conserved binding sites present upstream of each gene.

### The motif acts as a regulatory element

**Figure 5:**
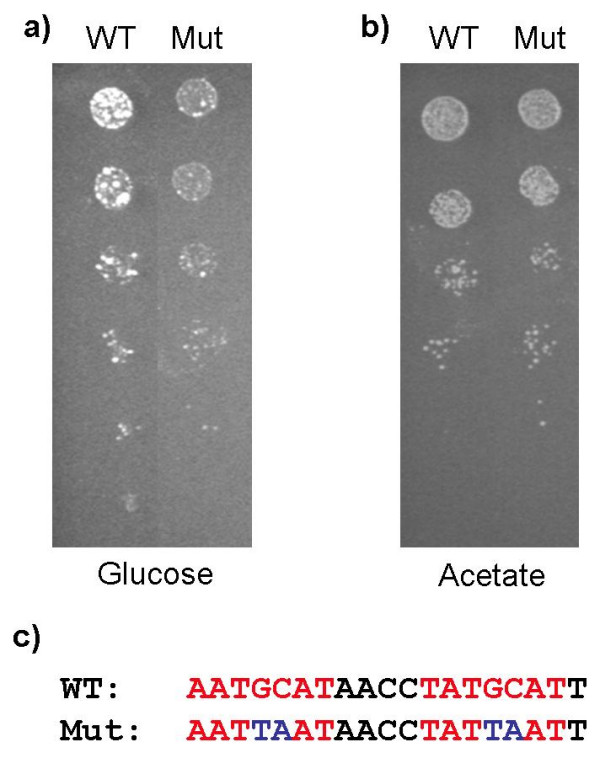
Activation of the reporter gene through the putative motif. **(a) **Growth on glucose containing 2 mM 3AT with the wild type motif (left) and the mutant motif (right) upstream of the reporter. **(b) **Growth on acetate containing 0.5 mM 3AT with the wild type motif (left) and the mutant motif (right) upstream of the reporter. **(c) **Sequences used for the reporter assay. Motifs are shown in red and mutations are shown in blue.

These findings lead us to hypothesize that our putative motif is a regulatory element important in modulating expression in the presence of different carbon sources. To test this hypothesis we inserted a 19 base pair sequence (shown in figure 5C) containing two conserved instances of the motif upstream of a *HIS3 *reporter gene. We also inserted a mutant version of the 19 base pair sequence (figure 5C) in front of a *HIS3 *reporter gene. To determine if our motif regulates expression we compared the growth of a strain containing the wild type sequence versus the mutant sequence in the presence of 3AT (3-amino triazole), an inhibitor of the *HIS3 *gene product. When plated on media containing glucose a substantial growth advantage was observed in the strain carrying a plasmid with the motif insert as compared to the strain carrying a plasmid with the mutant insert (figure 5A,B as well as compared to a strain carrying a plasmid with no insert (data not shown). However, when plated on media containing acetate as the sole carbon source the growth advantage is no longer observed. These results suggest that this motif activates expression of the reporter gene in the presence of glucose, but not in the presence of acetate. We conclude that this motif is a cis-regulatory element that responds to different carbon sources.

## Discussion

As the power of comparative genomics to find functional DNA elements becomes more apparent it is important to create practical statistical methods for describing evolutionary conservation. To this aim we developed a statistical test to assess transcription factor binding site conservation. The method takes into account the positional variation of explicit binding site models and weights significance in proportion to the evolutionary distances of the species under consideration. Based on simulations, the approach should be sensitive and specific when applied to commonly used sets of related species. In practice, the approach led to the discovery and subsequent validation of a functional regulatory element, thus proving the utility of the new approach on real data.

By defining an explicit model of TFBS evolution, we have taken an approach that is fundamentally different from recent large-scale studies in the field. We believe this is the primary reason why none of our motif predictions match the numerous predictions made by Kellis et al. [10], Cliften et al. [11] and Harbison et al. [28]. The motifs we have predicted are different from those identified in previous studies, but, are of high quality as shown by our experimental validation of a regulatory element. Therefore this method will be useful in conjunction with other commonly used computational approaches.

In this study we presented a whole genome motif finding algorithm that incorporates phylogenetic information. Other motif finding methods that incorporate phylogeny have used either expectation maximization or Gibbs sampling. We performed an exhaustive search to look for regulatory elements across the whole genome. By searching exhaustively we have omitted position specific variation in the first step of motif finding thus limiting the space of sequence patterns evaluated. There is a tradeoff between searching exhaustively and incorporating position specific variation. In this study, we decided to search exhaustively. It would be interesting to expand our method to incorporate degeneracy into the search algorithm.

The regulatory motif we identified does not match any entries in the widely used SCPD [29] or Transfac [30] databases. The motif is also not correlated with any motifs identified in large scale comparative genomic studies [10,11] or high-throughput Chromatin Immunoprecipitation studies [28,31] of yeast transcription factors. However, after the completion of this work an extensive literature search revealed one study in which a regulatory motif similar to the one we identified was identified by site-directed mutagenesis of the *PDC1 *promoter [32]. In agreement with our results this study also demonstrated that the motif is responsive to changes in carbon source and likely regulates the expression of genes involved in glycolysis. Our findings support the conclusion that this motif is indeed an important regulatory element, and warrants the inclusion of this cis-regulatory element in commonly used databases of known, validated regulatory motifs.

## Conclusion

We have developed a motif discovery method based on the principle that functional DNA sequences evolve in a different pattern than selectively neutral sequences. The approach is applicable to whole genome sequences and takes into account the specific phylogeny of the species under consideration. While half of the motif predictions made by our method match known regulatory elements, the novel motif predictions made by our algorithm are unique. We validated one motif prediction experimentally, showing that it is responsive to different carbon sources. The combination of the computational and experimental results suggests that our motif discovery method is a useful addition to the current suite of tools for finding regulatory elements.

## Methods

### Sequence data

The alignments used in this study were constructed from the intergenic regions of the species *S. cerevisiae*, *S. paradoxus*, *S. mikatae*, and *S. bayanus *[10]. To avoid using regions that were missing sequence data, or were of low quality, we only considered alignments that contained less than 50% indels in every species. This removed approximately one quarter of the total intergenic regions. To obtain the synonymous rate tree we used the PAML package[33] to perform a maximum likelihood estimation of the transition/transversion ratio, the branch lengths and the unrooted tree structure based on five randomly selected genes.

### Binding site matrices

The previously identified binding site matrices were obtained from the AlignACE[34,35] homepage [36]. The alignments obtained from the AlignACE homepage were converted into position specific count matrices.

### HKY85 model

In the HKY85 nucleotide substitution model, different equilibrium base frequencies are allowed, and transitions and transversions can occur at different rates [19]. The probability of observing a substitution from base *i *to base *j *at time *t *is:

Pij(t)={πj+πj(1θj−1)e−μt+(θj−πjθj)e−μtAi=jπj+πj(1θj−1)e−μt−(πjθj)e−μtAi≠j,transitionπj(1−e−μt)i≠j,transversion
 MathType@MTEF@5@5@+=feaafiart1ev1aaatCvAUfKttLearuWrP9MDH5MBPbIqV92AaeXatLxBI9gBaebbnrfifHhDYfgasaacH8akY=wiFfYdH8Gipec8Eeeu0xXdbba9frFj0=OqFfea0dXdd9vqai=hGuQ8kuc9pgc9s8qqaq=dirpe0xb9q8qiLsFr0=vr0=vr0dc8meaabaqaciaacaGaaeqabaqabeGadaaakeaacqWGqbaudaWgaaWcbaGaemyAaKMaemOAaOgabeaakiabcIcaOiabdsha0jabcMcaPiabg2da9maaceaabaqbaeqabmGaaaqaaiabec8aWnaaBaaaleaacqWGQbGAaeqaaOGaey4kaSIaeqiWda3aaSbaaSqaaiabdQgaQbqabaGcdaqadaqaamaalaaabaGaeGymaedabaGaeqiUde3aaSbaaSqaaiabdQgaQbqabaaaaOGaeyOeI0IaeGymaedacaGLOaGaayzkaaGaemyzau2aaWbaaSqabeaacqGHsislcqaH8oqBcqWG0baDaaGccqGHRaWkdaqadaqaamaalaaabaGaeqiUde3aaSbaaSqaaiabdQgaQbqabaGccqGHsislcqaHapaCdaWgaaWcbaGaemOAaOgabeaaaOqaaiabeI7aXnaaBaaaleaacqWGQbGAaeqaaaaaaOGaayjkaiaawMcaaiabdwgaLnaaCaaaleqabaGaeyOeI0IaeqiVd0MaemiDaqNaemyqaeeaaaGcbaGaemyAaKMaeyypa0JaemOAaOgabaGaeqiWda3aaSbaaSqaaiabdQgaQbqabaGccqGHRaWkcqaHapaCdaWgaaWcbaGaemOAaOgabeaakmaabmaabaWaaSaaaeaacqaIXaqmaeaacqaH4oqCdaWgaaWcbaGaemOAaOgabeaaaaGccqGHsislcqaIXaqmaiaawIcacaGLPaaacqWGLbqzdaahaaWcbeqaaiabgkHiTiabeY7aTjabdsha0baakiabgkHiTmaabmaabaWaaSaaaeaacqaHapaCdaWgaaWcbaGaemOAaOgabeaaaOqaaiabeI7aXnaaBaaaleaacqWGQbGAaeqaaaaaaOGaayjkaiaawMcaaiabdwgaLnaaCaaaleqabaGaeyOeI0IaeqiVd0MaemiDaqNaemyqaeeaaaGcbaGaemyAaKMaeyiyIKRaemOAaOMaeiilaWIaemiDaqNaemOCaiNaemyyaeMaemOBa4Maem4CamNaemyAaKMaemiDaqNaemyAaKMaem4Ba8MaemOBa4gabaGaeqiWda3aaSbaaSqaaiabdQgaQbqabaGcdaqadaqaaiabigdaXiabgkHiTiabdwgaLnaaCaaaleqabaGaeyOeI0IaeqiVd0MaemiDaqhaaaGccaGLOaGaayzkaaaabaGaemyAaKMaeyiyIKRaemOAaOMaeiilaWIaemiDaqNaemOCaiNaemyyaeMaemOBa4Maem4CamNaemODayNaemyzauMaemOCaiNaem4CamNaemyAaKMaem4Ba8MaemOBa4gaaaGaay5Eaaaaaa@BCFA@

where π_*j *_represents the background or equilibrium frequency of base *j*, μ is the mutation rate, and θ_*j *_represents the total purine or pyrimidine frequency depending on base *j *(for instance, if *j *= G then θ_*j *_= π_G _+ π_*A*_). *A *is equal to θ_*j*_(κ - 1) + 1, where κ is the ratio of transitions to transversions. An HKY85 model has 4 parameters to estimate (3 base frequencies and κ).

### Parameter estimation for the conservation model

In order to model the conservation of a TFBS while incorporating position specific motif variation as well as phylogenetic information we apply a position specific HKY85 model of evolution. In this case the equilibrium frequencies match the frequencies of a particular position of a motif matrix after a small pseudocount is added. The neutral substitution rate (μt) (see Additional file 3) and the transition/transversion ratio (κ = 4.71) used in this model are estimated from synonymous sites and thought to be close to the true neutral substitution rate and transition/transversion ratio.

This model assumes that if the functionality of a binding site is preserved, then mutations are only tolerated in degenerate positions of the motif. Implicit in this assumption is that the amount of conservation and the importance for functionality are correlated at each position in the motif, which has been examined and supported in [37]. In some instances, such as the Swi5 binding site (G/T)GCTG(A/G)[29], there are only a few tolerated mutations at somewhat degenerate positions. By using a model that allows for these mutations, we can accurately assay the conservation of a binding site.

### Parameter estimation for the neutral model

To query a binding site for conservation, we compare how well the orthologous sequence fits a neutral (null) model and a conserved model. In the case of the neutral model an HKY85 model is used with the equilibrium frequencies equal to the background genomic frequencies (p(A) = p(T) = 0.31). Again the neutral substitution rate and the transition/transversion ratio used in this model are estimated from synonymous sites.

### Likelihood comparison

To assess the fit of the conserved model versus the neutral model we perform a likelihood comparison, which is very similar to a likelihood ratio test. The difference being that a likelihood ratio test requires maximum likelihood estimates of each model's parameters, while in our likelihood comparison the parameters are treated as input and not optimized. To perform the comparison, the likelihood function of the sequence alignment is calculated for the conserved model and the neutral model. The likelihood function *L *is calculated at each internal node that is connected to two leaves of the phylogenetic tree as:

P(sequence|model)=L=∏i=1w∑b=ATP(Xi|Ab,πi)P(Yi|Ab,πi)P(Ab)
 MathType@MTEF@5@5@+=feaafiart1ev1aaatCvAUfKttLearuWrP9MDH5MBPbIqV92AaeXatLxBI9gBaebbnrfifHhDYfgasaacH8akY=wiFfYdH8Gipec8Eeeu0xXdbba9frFj0=OqFfea0dXdd9vqai=hGuQ8kuc9pgc9s8qqaq=dirpe0xb9q8qiLsFr0=vr0=vr0dc8meaabaqaciaacaGaaeqabaqabeGadaaakeaacqWGqbaucqGGOaakcqqGZbWCcqqGLbqzcqqGXbqCcqqG1bqDcqqGLbqzcqqGUbGBcqqGJbWycqqGLbqzcqGG8baFcqqGTbqBcqqGVbWBcqqGKbazcqqGLbqzcqqGSbaBcqGGPaqkcqGH9aqpcqWGmbatcqGH9aqpdaqeWbqaamaaqahabaGaemiuaaLaeiikaGIaemiwaG1aaSbaaSqaaiabdMgaPbqabaGccqGG8baFcqWGbbqqdaWgaaWcbaGaemOyaigabeaakiabcYcaSiabec8aWnaaBaaaleaacqWGPbqAaeqaaOGaeiykaKIaemiuaaLaeiikaGIaemywaK1aaSbaaSqaaiabdMgaPbqabaGccqGG8baFcqWGbbqqdaWgaaWcbaGaemOyaigabeaakiabcYcaSiabec8aWnaaBaaaleaacqWGPbqAaeqaaOGaeiykaKIaemiuaaLaeiikaGIaemyqae0aaSbaaSqaaiabdkgaIbqabaGccqGGPaqkaSqaaiabdkgaIjabg2da9iabdgeabbqaaiabdsfaubqdcqGHris5aaWcbaGaemyAaKMaeyypa0JaeGymaedabaGaem4DaChaniabg+Givdaaaa@74DA@

where *X*_*i *_and *Y*_*i *_are the observed bases at position *i *in the motif alignment from different species, *A*_*b *_is the unobserved ancestral base, *w *is the length of the motif and *P*(*A*_*b*_) is the probability that the ancestral sequence is *A*_*b *_which is equal to the equilibrium base frequencies. The probabilities *P*(*X*_*i *_| *A*_*b*_, π_*i*_) come from the HKY85 model described above. This setup is extended to cover the entire tree using Felsenstein's pruning algorithm [18].

### Threshold determination for likelihood comparison

The statistic calculated for the likelihood comparison is:

Λ=L(conserved)L(neutral)
 MathType@MTEF@5@5@+=feaafiart1ev1aaatCvAUfKttLearuWrP9MDH5MBPbIqV92AaeXatLxBI9gBaebbnrfifHhDYfgasaacH8akY=wiFfYdH8Gipec8Eeeu0xXdbba9frFj0=OqFfea0dXdd9vqai=hGuQ8kuc9pgc9s8qqaq=dirpe0xb9q8qiLsFr0=vr0=vr0dc8meaabaqaciaacaGaaeqabaqabeGadaaakeaacqqHBoatcqGH9aqpdaWcaaqaaiabdYeamjabcIcaOiabdogaJjabd+gaVjabd6gaUjabdohaZjabdwgaLjabdkhaYjabdAha2jabdwgaLjabdsgaKjabcMcaPaqaaiabdYeamjabcIcaOiabd6gaUjabdwgaLjabdwha1jabdsha0jabdkhaYjabdggaHjabdYgaSjabcMcaPaaaaaa@4AF5@

Under certain assumptions for a likelihood ratio test, the quantity 2 ln(λ) is approximately Chi-squared distributed under the null (neutral) hypothesis [20]. The number of degrees of freedom for the Chi-squared distribution is equal to the difference in the number of free parameters of the two models. Since κ is the same for each model, the conserved model has 3 free parameters, referring to the base frequencies, at each column of the binding site, where the neutral model has three free parameters for the entire binding site. Therefore the number of degrees of freedom for the Chi-squared distribution is equal to 3 * (*w *- 1).

Since the base frequencies for both the neutral and conserved models are treated as input and not optimized, we are performing a likelihood comparison and not a likelihood ratio test. For the likelihood comparison there is no expected distribution under the null model. To empirically determine a cutoff we simulated every hexamer neutrally evolving 1000 times using the phylogeny for the yeast species under consideration (see Additional file 3). For each simulated sequence we calculated ln(λ), and combined every observation to create an overall null distribution for ln(λ) (see Additional file 4). We determined the 0.1% false positive rate cutoff for ln(λ) to be 17.71. This cutoff is similar to the cutoff calculated for a likelihood ratio test (18.84) using a Chi-squared distribution as the null distribution for 2*ln(λ). We performed the same analysis for all pentamers (five base pair sequences) and heptamers (seven base pair sequences) (see Additional file 4). In each case the empirically determined cutoff is similar to and slightly lower than the Chi-squared calculated cutoff (14.61 vs. 16.45 for pentamers, 18.01 vs. 21.16 for heptamers). Based on these simulations we decided to use the Chi-squared estimate for significance, as is done with likelihood ratio tests, because it is generally applicable to motifs of different sizes and is a conservative measure of significance.

### Motif conservation simulations

Simulations to determine the sensitivity and specificity of the likelihood comparison were performed as follows. An ancestral intergenic sequence of 1000 base pairs was constructed which contained one instance of the motif. For the simulations we made the simplifying assumption that the underlying phylogeny had a star topology with equal branch lengths. For the non-motif sequence, substitutions were introduced according to the neutral model described above. For the conservation simulations, the conserved model was used to introduce substitutions into the motif. For the non-conserved simulations the first species created was constrained to retain the motif, while in the other species the motif section evolved under the neutral model. For each set of parameters (number of species and evolutionary distance), five known motifs were used (Rgt1, Rap1, Cbf1, Abf1 and Leu3). For each motif and set of parameters 1000 different sequences were simulated. After simulating the sequences, we used the program Patser [38] with the automatically calculated cutoff to find all instances of the motif and then tested these sites using the likelihood comparison described above with a 99.9% confidence cutoff. To determine the evolutionary distance of commonly used organisms, we added up the branch lengths of synonymous rate trees and then divided by the number of species.

### Testing hexamers

In order to determine which six basepair sequences are significantly conserved we tested all hexamers for having a significant number of conserved sites. For each hexamer, each instance was found in the intergenic regions of the *S. cerevisiae *genome and the likelihood comparison was performed as described above with a 99.9% confidence cutoff. For the likelihood comparison, the conserved model used a binding site matrix with 100.25 counts for the correct base at a particular position and 0.25 for the incorrect base at a particular position. Using this method the total number of instances and the number of conserved instances were calculated for each hexamer. Then a Chi-squared test was performed with one degree of freedom, where the expected number of conserved instances was equal to the overall frequency of conserved hexamers (6.97%) times the total number of instances for each hexamer. A 99.9% confidence level was chosen as significant. Hexamers were considered to match known motifs if they had a correlation coefficient of greater than 0.7 (computed using CompareACE [35]).

### Combining significant hexamers

To create motifs from significant hexamers, we tried to align each pair of hexamers in the best way possible. We seeded an alignment with one hexamer and combined every hexamer that matched at 4 positions or more to the alignment seed. After each significant hexamer had seeded an alignment, we compared the alignments using CompareACE [35] and combined any alignments that had a correlation coefficient greater than 0.7.

### Reporter assay

For the reporter assays, we used PJ69-4α (*MATα trp1-901 leu2-3,112 ura3-52 his3-200 gal4 gal80 LYS2::GAL1-HIS3 GAL2-ADE2 met2::GAL7-lacZ*) to carry the reporter plasmids [39]. In order to create the reporter plasmids a *HIS3 *reporter plasmid pBM4429 (backbone CEN plasmid with *URA3*, [9,40]) was cut with Spe1 and Xho1 and gel-purified for gap repair with the double stranded motif (sequences shown in figure 5C). Cells carrying the plasmid were grown on restrictive media to an OD_600 _= 1.0 and 10 fold dilutions were subsequently plated on media containing either 2% glucose or 2% potassium acetate and varying concentrations of 3AT.

## Authors' contributions

JG implemented the computational approach and performed the experiments. BC and JG drafted the manuscript. All authors conceived and designed the study.

## Supplementary Material

Additional File 1 Significantly conserved hexamers, Lists all hexamers that are conserved a significant fraction of the time. Click here for file

Additional File 2 Binding Site Predictions, Lists all count matrices for binding sites predictions that do not match known sites. Click here for file

Additional File 3 Yeast Phylogenetic Tree, Synonymous rate tree used, branch lengths are measured in number of substitutions per site. Click here for file

Additional File 4 Positive tails of the distributions of ln(λ) for neutrally evolving simulation of all pentamers (A), hexamers (B) and heptamers (C). Click here for file

## References

[B1] Korf I, Flicek P, Duan D, Brent MR (2001). Integrating genomic homology into gene structure prediction. Bioinformatics.

[B2] Wiehe T, Gebauer-Jung S, Mitchell-Olds T, Guigo R (2001). SGP-1: prediction and validation of homologous genes based on sequence alignments. Genome Res.

[B3] Coventry A, Kleitman DJ, Berger B (2004). MSARI: multiple sequence alignments for statistical detection of RNA secondary structure. Proc Natl Acad Sci U S A.

[B4] Rivas E, Eddy SR (2001). Noncoding RNA gene detection using comparative sequence analysis. BMC Bioinformatics.

[B5] Washietl S, Hofacker IL, Stadler PF (2005). Fast and reliable prediction of noncoding RNAs. Proc Natl Acad Sci U S A.

[B6] Wang T, Stormo GD (2003). Combining phylogenetic data with co-regulated genes to identify regulatory motifs. Bioinformatics.

[B7] Loots GG, Ovcharenko I, Pachter L, Dubchak I, Rubin EM (2002). rVista for comparative sequence-based discovery of functional transcription factor binding sites. Genome Res.

[B8] Blanchette M, Schwikowski B, Tompa M (2002). Algorithms for phylogenetic footprinting. J Comput Biol.

[B9] Gertz J, Riles L, Turnbaugh P, Ho SW, Cohen BA (2005). Discovery, validation, and genetic dissection of transcription factor binding sites by comparative and functional genomics. Genome Res.

[B10] Kellis M, Patterson N, Endrizzi M, Birren B, Lander ES (2003). Sequencing and comparison of yeast species to identify genes and regulatory elements. Nature.

[B11] Cliften P, Sudarsanam P, Desikan A, Fulton L, Fulton B, Majors J, Waterston R, Cohen BA, Johnston M (2003). Finding functional features in Saccharomyces genomes by phylogenetic footprinting. Science.

[B12] Stormo GD (2000). DNA binding sites: representation and discovery. Bioinformatics.

[B13] Sinha S, Blanchette M, Tompa M (2004). PhyME: a probabilistic algorithm for finding motifs in sets of orthologous sequences. BMC Bioinformatics.

[B14] Moses AM, Chiang DY, Eisen MB (2004). Phylogenetic motif detection by expectation-maximization on evolutionary mixtures. Pac Symp Biocomput.

[B15] Siddharthan R, Siggia ED, van Nimwegen E (2005). PhyloGibbs: A Gibbs Sampling Motif Finder That Incorporates Phylogeny. PLoS Computational Biology.

[B16] Jukes THCRC, Munro HN (1969). Evolution of protein molecules. Mammalian protein metabolism.

[B17] Sinha S, van Nimwegen E, Siggia ED (2003). A probabilistic method to detect regulatory modules. Bioinformatics.

[B18] Felsenstein J (1981). Evolutionary trees from DNA sequences: a maximum likelihood approach. J Mol Evol.

[B19] Hasegawa M, Kishino H, Yano T (1985). Dating of the human-ape splitting by a molecular clock of mitochondrial DNA. J Mol Evol.

[B20] Huelsenbeck JP, Rannala B (1997). Phylogenetic methods come of age: testing hypotheses in an evolutionary context. Science.

[B21] Langley CH, Fitch WM (1974). An examination of the constancy of the rate of molecular evolution. J Mol Evol.

[B22] Navidi WC, Churchill GA, von Haeseler A (1991). Methods for inferring phylogenies from nucleic acid sequence data by using maximum likelihood and linear invariants. Mol Biol Evol.

[B23] Muse SV (1996). Estimating synonymous and nonsynonymous substitution rates. Mol Biol Evol.

[B24] Goldman N (1993). Statistical tests of models of DNA substitution. J Mol Evol.

[B25] Eddy SR (2005). A model of the statistical power of comparative genome sequence analysis. PLoS Biol.

[B26] Koch C, Moll T, Neuberg M, Ahorn H, Nasmyth K (1993). A role for the transcription factors Mbp1 and Swi4 in progression from G1 to S phase. Science.

[B27] Cho RJ, Campbell MJ, Winzeler EA, Steinmetz L, Conway A, Wodicka L, Wolfsberg TG, Gabrielian AE, Landsman D, Lockhart DJ, Davis RW (1998). A genome-wide transcriptional analysis of the mitotic cell cycle. Mol Cell.

[B28] Harbison CT, Gordon DB, Lee TI, Rinaldi NJ, Macisaac KD, Danford TW, Hannett NM, Tagne JB, Reynolds DB, Yoo J, Jennings EG, Zeitlinger J, Pokholok DK, Kellis M, Rolfe PA, Takusagawa KT, Lander ES, Gifford DK, Fraenkel E, Young RA (2004). Transcriptional regulatory code of a eukaryotic genome. Nature.

[B29] Zhu J, Zhang MQ (1999). SCPD: a promoter database of the yeast Saccharomyces cerevisiae. Bioinformatics.

[B30] Wingender E, Chen X, Fricke E, Geffers R, Hehl R, Liebich I, Krull M, Matys V, Michael H, Ohnhauser R, Pruss M, Schacherer F, Thiele S, Urbach S (2001). The TRANSFAC system on gene expression regulation. Nucleic Acids Res.

[B31] Lee TI, Rinaldi NJ, Robert F, Odom DT, Bar-Joseph Z, Gerber GK, Hannett NM, Harbison CT, Thompson CM, Simon I, Zeitlinger J, Jennings EG, Murray HL, Gordon DB, Ren B, Wyrick JJ, Tagne JB, Volkert TL, Fraenkel E, Gifford DK, Young RA (2002). Transcriptional regulatory networks in Saccharomyces cerevisiae. Science.

[B32] Liesen T, Hollenberg CP, Heinisch JJ (1996). ERA, a novel cis-acting element required for autoregulation and ethanol repression of PDC1 transcription in Saccharomyces cerevisiae. Mol Microbiol.

[B33] Yang Z (1997). PAML: a program package for phylogenetic analysis by maximum likelihood. Comput Appl Biosci.

[B34] Roth FP, Hughes JD, Estep PW, Church GM (1998). Finding DNA regulatory motifs within unaligned noncoding sequences clustered by whole-genome mRNA quantitation. Nat Biotechnol.

[B35] Hughes JD, Estep PW, Tavazoie S, Church GM (2000). Computational identification of cis-regulatory elements associated with groups of functionally related genes in Saccharomyces cerevisiae. J Mol Biol.

[B36] AlignACE Homepage. http://atlas.med.harvard.edu/.

[B37] Moses AM, Chiang DY, Kellis M, Lander ES, Eisen MB (2003). Position specific variation in the rate of evolution in transcription factor binding sites. BMC Evol Biol.

[B38] Hertz GZ, Stormo GD (1999). Identifying DNA and protein patterns with statistically significant alignments of multiple sequences. Bioinformatics.

[B39] James P, Halladay J, Craig EA (1996). Genomic libraries and a host strain designed for highly efficient two-hybrid selection in yeast. Genetics.

[B40] Sikorski RS, Hieter P (1989). A system of shuttle vectors and yeast host strains designed for efficient manipulation of DNA in Saccharomyces cerevisiae. Genetics.

[B41] Crooks GE, Hon G, Chandonia JM, Brenner SE (2004). WebLogo: a sequence logo generator. Genome Res.

