# Supplementary figures and images for: Phylogeny based discovery of regulatory elements
Source: BMC Bioinformatics. 2006 May 22;7:266. doi: 10.1186/1471-2105-7-266 (PMC1525002; doi:10.1186/1471-2105-7-266)

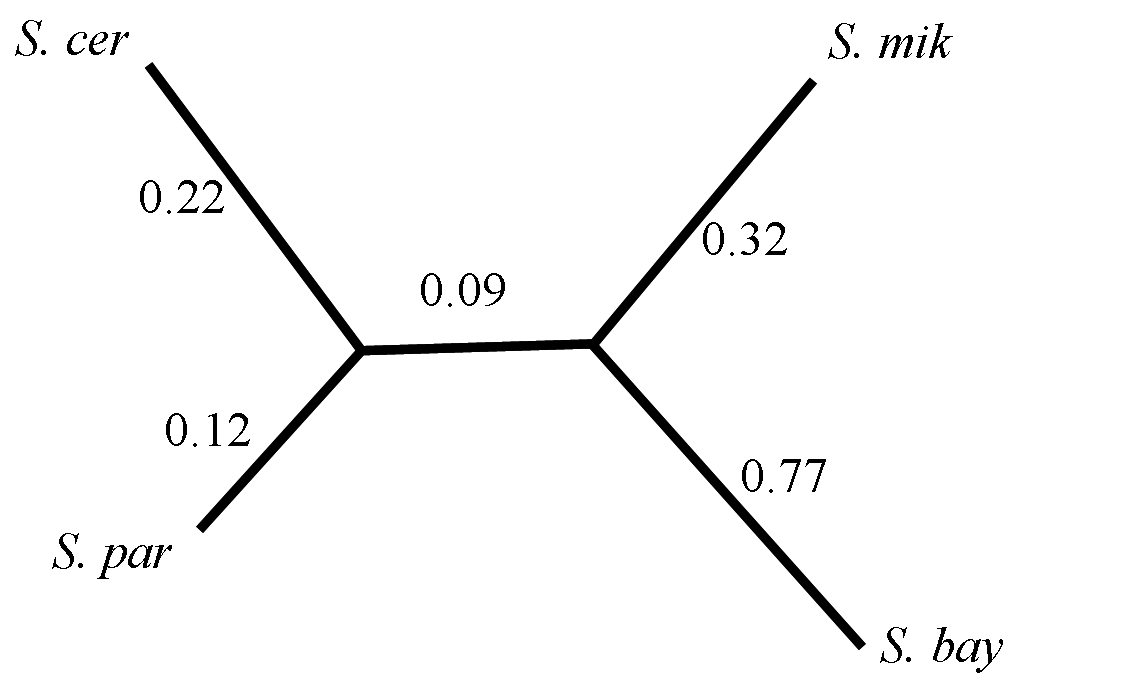

Supplement: Additional File 3 — Yeast Phylogenetic Tree, Synonymous rate tree used, branch lengths are measured in number of substitutions per site. [file 1471-2105-7-266-S1.tiff]

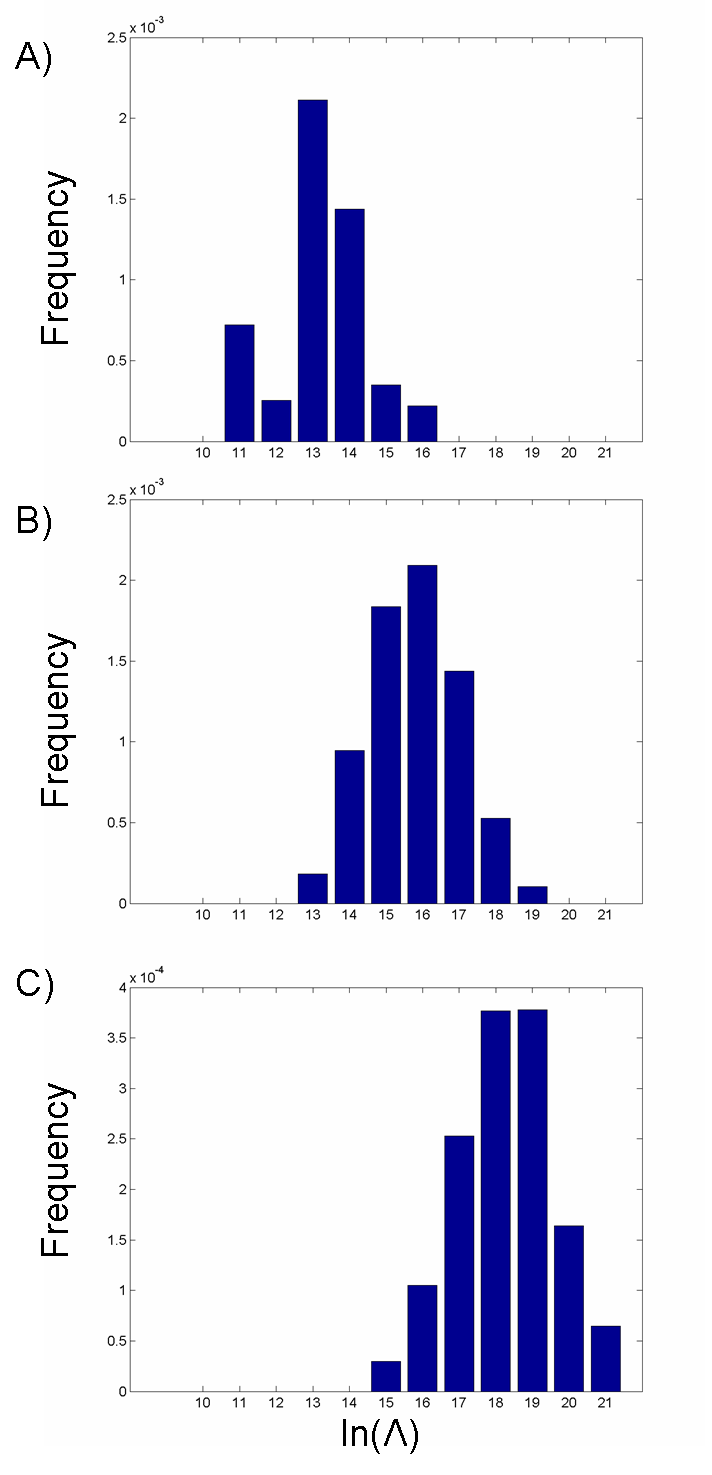

Supplement: Additional File 4 — Positive tails of the distributions of ln(λ) for neutrally evolving simulation of all pentamers (A), hexamers (B) and heptamers (C). [file 1471-2105-7-266-S4.tiff]
